# Disentangling global atrophy burden from local structural patterns reveals clinically relevant heterogeneity in mild cognitive impairment

**DOI:** 10.21203/rs.3.rs-9443570/v1

**Published:** 2026-05-11

**Authors:** Xiaoming Yang, Jian Lyu, Jubo Wang, Yu Quan

**Affiliations:** Second Affiliated Hospital of Xi’an Jiaotong University; Second Affiliated Hospital of Xi’an Jiaotong University; Second Affiliated Hospital of Xi’an Jiaotong University; Second Affiliated Hospital of Xi’an Jiaotong University

**Keywords:** mild cognitive impairment, structural MRI, Alzheimer’s disease, global atrophy index, residualization

## Abstract

**Background:**

Mild cognitive impairment (MCI) is a heterogeneous prodromal stage of Alzheimer’s disease (AD). Imaging-based subtyping studies, however, often confound overall disease severity with local anatomical variation, limiting biological interpretability and clinical utility. We aimed to separate global neurodegenerative burden from local structural patterns and examine their clinical relevance in MCI.

**Methods:**

Baseline ADNI-1 data from 731 participants were analyzed, including 205 cognitively normal individuals, 351 with MCI, and 175 with AD. A global atrophy index (GAI) was constructed from whole-brain gray matter volume, white matter volume, cerebrospinal fluid volume, and mean cortical thickness to quantify overall neurodegenerative burden. Candidate regions of interest were screened in the full sample. Within MCI, regional measures were residualized against age, sex, education, and GAI to derive local structural features independent of global atrophy. Principal component analysis and unsupervised clustering were then used to identify subtypes. Associations with cognition, daily functioning, and neuropsychiatric symptoms were assessed using multivariable linear regression, and nested models were compared to quantify incremental explained variance (ΔR^2^) from continuous local residual features beyond subtype labels.

**Results:**

The GAI showed a clear gradient across cognitively normal, MCI, and AD groups. After controlling for GAI, two reproducible local structural patterns were identified within MCI: a thalamic-subcortical subtype and a frontal-paralimbic subtype. Subtype differences remained associated with cognition and daily functioning after adjustment for demographic factors and GAI, whereas associations with neuropsychiatric symptoms were not significant or were markedly weaker. Adding PC-based continuous local residual features provided incremental explanatory value for ADAS-Cog13 and FAQ beyond models including subtype labels.

**Conclusions:**

MCI heterogeneity is not explained solely by global brain atrophy. Local structural patterns independent of overall neurodegenerative burden capture clinically meaningful variation and may improve imaging-based stratification in prodromal AD.

## Introduction

Alzheimer’s disease (AD) is the most common type of neurodegenerative dementia. Its main clinical features are progressive cognitive decline and reduced ability in daily living. It has become an important public health issue in the context of global aging.(1, 2) As research on disease-modifying treatment and early intervention continues to advance, growing evidence suggests that the timing of intervention largely depends on accurate identification of the predementia stage, especially precise detection of high-risk individuals among people with mild cognitive impairment (MCI).(3–5) However, MCI is not a homogeneous clinical state. Different individuals show marked differences in patterns of cognitive impairment, daily functioning, and rate of disease progression.(6, 7) This heterogeneity indicates that traditional clinical classification alone is not sufficient to fully describe individual differences in the prodromal stage of AD, which in turn limits the efficiency of cohort enrichment and precise stratification.

Structural magnetic resonance imaging (sMRI) is a key noninvasive tool for assessing AD-related neurodegeneration. As the disease progresses, sMRI can reveal a series of global changes, including enlargement of cerebrospinal fluid spaces, loss of gray and white matter volume, and cortical thinning.(8–10) Imaging-based quantitative measures built from these whole-brain markers can reflect the overall burden of neurodegeneration and are associated with clinical stage and severity of cognitive impairment.(11–13) Therefore, global atrophy measures are usually regarded as summary markers of disease severity, with good stability and comparability across samples.(14–16) Even so, clinical observations show that MCI individuals with similar overall levels of atrophy may still differ greatly in cognition and daily functioning.(17, 18) This suggests that the degree of global brain atrophy reflects only the total accumulated burden of disease and cannot fully explain the complex clinical differences seen in the prodromal stage.

To obtain more detailed anatomical information, many studies have turned to local structural changes in specific brain regions and have attempted to map these regions to impairment in specific cognitive domains.(19, 20) The problem is that local structural changes are often inseparable from the background of overall neurodegeneration. When the volume of a given brain region is reduced, it is often difficult to determine whether this reflects a local change that simply accompanies whole-brain atrophy, or a specific spatial pattern beyond the global burden, within most current analytic frameworks.(21, 22) This issue is especially prominent in imaging-based subtyping studies of MCI, particularly in unsupervised imaging subtyping research. Many previous clustering studies have directly used high-dimensional brain structural features to identify subtypes. As a result, their findings may have mixed gradients of disease severity with true differences in local spatial patterns, which weakens both the biological basis of the subtypes and their clinical value.(23–25)

The key issue in current research is how to characterize both the global burden of neurodegeneration and local brain structural patterns within the same analytic framework, while statistically separating their mutual effects. This study aims to answer three questions. First, we aim to determine whether stable differences in local brain structural patterns still exist within individuals with MCI after controlling for the overall burden of brain atrophy. Second, we seek to evaluate whether these local differences are independently associated with cognitive and functional outcomes. Third, we investigate whether continuous local structural information can provide additional clinical value beyond discrete subtype labels. To address these objectives, we propose a hierarchical structural analysis strategy from global to local levels. First, we integrate whole-brain structural measures across the full AD spectrum sample to construct a global atrophy index (GAI), which quantifies the overall burden of degeneration. Next, within the MCI subgroup, candidate brain regions are residualized to extract local structural features independent of the GAI, followed by dimensionality reduction and unsupervised clustering. Finally, we examine the associations of the resulting subtypes and local features with clinical scales.

This study proposes the following hypotheses. First, the constructed GAI can accurately capture the overall neurodegenerative gradient across the continuum from cognitively normal individuals to those with MCI and AD. Second, stable imaging subtypes with clear anatomical meaning can be identified in the MCI population based on local residual features. Third, these local patterns, independent of global atrophy, are not only associated with cognitive and functional status, but can also provide more refined information on individual differences beyond conventional subtype stratification.

## Methods

### 2.1 Study participants

Baseline data for this study were obtained from the Alzheimer’s Disease Neuroimaging Initiative (ADNI), focusing on participants in the ADNI-1 cohort.(26) This database systematically collects clinical and multimodal imaging data from participants. A total of 731 participants were finally included in this study, including 205 cognitively normal (CN) individuals, 351 individuals with MCI, and 175 individuals with AD. According to established criteria, participants in the CN group were required to have no clear memory complaint, a Clinical Dementia Rating (CDR)(27) global score of 0, overall cognitive performance within the normal range, largely preserved daily functioning, and no diagnosis of MCI or dementia. Participants in the MCI group were required to have a subjective memory complaint, relatively preserved overall cognitive function, largely intact daily functioning, a CDR global score of 0.5, and no diagnosis of dementia. Participants in the AD group were required to meet the the National Institute of Neurological and Communicative Disorders and Stroke and the Alzheimer's Disease and Related Disorders Association (NINCDS/ADRDA) criteria for probable AD,(28) with clear cognitive and functional impairment.

### 2.2 Demographic, clinical, and genetic variables

Demographic variables included age, sex, and years of education. Clinical phenotypes were assessed using the Alzheimer’s Disease Assessment Scale-Cognitive Subscale (ADAS-Cog13)(29) for overall cognitive performance, the Functional Activities Questionnaire (FAQ)(30) for daily functioning, and the Neuropsychiatric Inventory (NPI)(31) for behavioral and psychiatric symptoms. Genetic status was classified according to the presence or absence of the apolipoprotein E *ε4* (*APOE ε4*) allele.

### 2.3 Structural MRI measures and the global atrophy index

This study used baseline 1.5T T1-weighted structural MRI data provided by the ADNI database. Image preprocessing was performed in a unified manner in MATLAB R2023a with the Computational Anatomy Toolbox (CAT12 v12.8).(32) To accurately extract morphological features from each brain region, the preprocessing pipeline included the following steps. First, denoising and N4 bias field correction were applied to reduce signal inhomogeneity caused by scanning artifacts. Next, individual images were normalized to the MNI 152 template with a voxel resolution of 2 mm × 2 mm × 2 mm using the DARTEL algorithm.(33, 34) This algorithm improves cross-subject registration by carefully handling anatomical variation between individuals.(34) Finally, the images were automatically segmented into gray matter, white matter, and cerebrospinal fluid, and the corresponding tissue volumes and cortical thickness measures were extracted.

For local region-of-interest (ROI) volume features, this study used the residual method to adjust for total intracranial volume (TIV),(35) in order to remove the confounding effect of differences in head size. The values corrected by the residual method were directly used in later feature analysis. The formula was:

$$ROI_{adj}=residuals\left[lm\left(ROI∼TIV\right)\right]+mean\left(ROI\right)$$

To quantitatively describe the overall burden of brain atrophy in each individual, this study further integrated whole-brain macrostructural measures and constructed a global atrophy index (GAI). To quantify differences in overall degenerative burden across subjects along the disease continuum, the GAI was defined as:

$$GAI=Z\left(CSF_{adj}\right)-Z\left(GM_{adj}\right)-Z\left(WM_{adj}\right)-Z\left(Thickness\right)$$

CSFadj, GMadj, and WMadj denote total CSF/GM/WM volumes adjusted for intracranial volume using residual approach. Thickness denotes mean cortical thickness averaged across the whole cortex. Z-standardization was performed across the full sample. It integrates four widely used whole-brain structural indicators that capture complementary aspects of tissue loss and brain atrophy: cerebrospinal fluid expansion, gray matter loss, white matter loss, and cortical thinning. Rather than optimizing prediction for any single outcome, the purpose of the GAI was to provide an interpretable and sample-comparable summary metric of global structural degeneration across the AD continuum. Equal weighting was adopted after z-standardization to avoid overfitting and to preserve transparency of interpretation, so that higher GAI values consistently reflect greater whole-brain atrophic burden. In the present framework, the GAI was not intended to replace regional measures, but to serve as a parsimonious global reference against which local structural deviations could be estimated. A higher GAI value indicates more severe overall brain atrophy and neurodegeneration. In later models, GAI was used as a key covariate to adjust for the effect of overall atrophy.

### 2.4 Data quality control and missing data handling

After the original imaging features were obtained, this study first combined the volumes of homologous regions in the left and right hemispheres to reduce redundancy in the high-dimensional feature space and to focus on overall structural changes in bilateral systems. The combined data then underwent systematic quality assessment. Because unsupervised clustering algorithms are highly sensitive to noise and missing values in high-dimensional feature space, this study used a strict feature-level screening strategy.

During automatic image segmentation, some brain regions may be affected by individual anatomical variation or local susceptibility artifacts, which can lead to failed feature extraction or low confidence. To avoid statistical bias caused by manual imputation of a large number of missing values, this study set a strict retention threshold and directly removed local brain regions with more than 10% missing values in the full sample. After this step, 26 brain region features were excluded.

This strict quality control step minimized algorithmic noise as much as possible, while the retained feature set still covered the core cortical and subcortical anatomical networks closely related to the pathological process of AD. After feature-level screening, for the very small number of randomly missing individual data points, this study did not use imputation. Instead, complete-case analysis was performed within each model to ensure internal consistency and data matching in each analysis module.

### 2.5 Candidate ROI screening in the full sample

To ensure that the brain structural features entering the within-MCI analysis had clear disease relevance and to avoid introducing high-dimensional redundant noise, this study first screened candidate brain regions at the full-sample level. A general linear model was used to test differences in each ROI among the CN, MCI, and AD groups, with age, sex, and years of education as covariates. The main effect of disease group was extracted, and false discovery rate (FDR) correction was performed using the Benjamini-Hochberg method. Brain regions retained after this step were defined as candidate ROIs related to disease stage and were used as input for later local feature engineering in the MCI group.

### 2.6 Construction of local residual features in MCI

To separate the effect of overall atrophy on local brain regions, linear regression models were built within the MCI subgroup. The values of the selected candidate ROIs were used as dependent variables, and age, sex, years of education, and GAI were used as independent variables. The residuals from each model were extracted and defined as local residual features. These features represent the degree to which the structure of a specific brain region deviates from the expected mean in individuals with similar age background and similar global neurodegenerative burden. After residualization, the features were standardized and principal component analysis (PCA) was performed. Principal components were retained until the cumulative explained variance reached 80%, in order to reduce dimensionality and suppress noise between features.

### 2.7 Imaging subtype identification and stability testing

Unsupervised clustering was performed in the MCI sample using the principal components after dimensionality reduction. Several algorithms were examined, including K-means, Gaussian mixture models, hierarchical clustering, and partitioning around medoids, with 2 to 5 candidate cluster numbers tested. The final solution was selected by jointly considering the silhouette coefficient, Gap statistic, Bayesian information criterion, and the balance of cluster size distribution. To assess the reliability of the clustering results, Bootstrap resampling was used to calculate the adjusted rand index and the mean Jaccard index. Consistency across different clustering algorithms was also examined.

### 2.8 Clinical association and analysis of additional explanatory value

After subtype labels were obtained, baseline differences in clinical and demographic variables across subtypes were assessed using analysis of variance (ANOVA) for continuous variables and the chi-square test for categorical variables. Multivariable linear regression models were then fit with ADAS-Cog13, FAQ, and NPI as outcome variables. To test the main effect of subtype while accounting for potential confounding, models included age, sex, and years of education and additionally adjusted for global atrophy index (GAI).

To evaluate whether continuous local structural features provided explanatory value beyond discrete subtype labels, we compared a sequence of nested models within the MCI subgroup: (i) a covariate-only base model (age, sex, education, and GAI), (ii) a subtype-augmented model (base + subtype), and (iii) an extended model that further included principal components (PCs) derived from local residual structural features (base + subtype + PCs; k = 24, explaining ≥ 80% cumulative variance). Model explanatory performance was summarized using R^2^ and adjusted R^2^ and incremental contributions (ΔR^2^) were evaluated with nested-model F tests; p-values for the extended-vs-subtype step were corrected across outcomes using the Benjamini–Hochberg FDR procedure.

## Results

### 3.1 Baseline characteristics and distribution of the global imaging gradient

A total of 731 participants were included in this study, including 205 cognitively normal controls, 351 patients with mild cognitive impairment, and 175 patients with Alzheimer’s disease. Detailed baseline data are shown in Table 1. There were no significant differences among the three groups in age or total intracranial volume. Significant group differences were found in sex distribution, years of education, and *APOE ε4* carrier status, with the AD group showing the highest APOE ε4 carrier rate. For clinical measures, ADAS-Cog13 and FAQ scores showed a gradual worsening trend along the disease continuum. At the level of global imaging features, the GAI showed a clear stratification effect across groups, with the lowest mean burden in the CN group, an intermediate level in the MCI group, and the highest level in the AD group (F = 87.65, *p* < 0.001,, η^2^ = 0.147). At the same time, gray matter volume and mean cortical thickness also decreased significantly as the disease progressed. These findings confirm a consistent severity gradient across the full sample in both cognitive function and overall neurodegeneration.

### 3.2 Screening of candidate local brain regions in the full sample

Based on a general linear model with covariate adjustment and FDR correction for multiple testing, a total of 27 candidate ROIs with a significant main effect of disease group were identified in the full sample. These brain regions were widely distributed across the medial temporal system, thalamic and basal ganglia-related nuclei, and some frontal regions. In the three-group comparison, the hippocampal region showed highly significant between-group differences, and the anterior thalamic nuclei and putamen also showed strong associations with disease stage. These results confirm that changes in local brain structure occur across all stages of the AD spectrum (Table 2), and they provide the feature basis for later analyses aimed at removing the confounding effect of disease severity and exploring true heterogeneity within MCI.

### 3.3 Identification of local structural subtypes in MCI and their brain structural features

Within the MCI subgroup, residuals of the candidate ROIs were extracted after adjustment for age, sex, years of education, and GAI, and principal component analysis was then performed (Table 3). The retained principal components were entered into unsupervised clustering. Based on internal quality metrics and consistency across algorithms, the two-cluster solution derived from K-means showed the best overall stability and interpretability. To confirm subtype independence from global atrophy, GAI was compared between subgroups: no significant difference was observed (F = 0.307, p = 0.540, η^2^ = 0.003). Bootstrap resampling further supported the reproducibility of this two-cluster solution.

**Figure 1:**
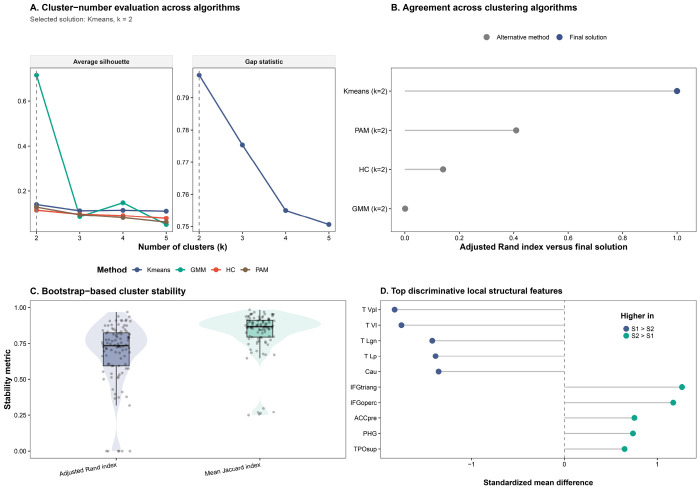
Identification and robustness of MCI imaging subtypes (A) Average silhouette values across K-means, GMM, HC, and PAM for candidate cluster numbers (k = 2–5), together with the K-means gap statistic. The dashed line marks the selected solution (k = 2). (B) Agreement between the final clustering solution and alternative clustering algorithms, quantified by ARI. (C) Bootstrap-based stability of the final two-cluster solution, summarized using ARI and mean Jaccard index across resamples. (D) Top local structural features discriminating the two MCI subtypes, ranked by SMD. Point color indicates the subtype with the higher value for each feature. Abbreviations: MCI, mild cognitive impairment; GMM, Gaussian mixture modeling; HC, hierarchical clustering; PAM, partitioning around medoids; ARI, adjusted Rand index; SMD, standardized mean difference.HIP, hippocampus; PHG, parahippocampal gyrus; tMGN, medial geniculate nucleus of the thalamus; tPuA, anterior pulvinar nucleus of the thalamus; AMY, amygdala; mOFC, medial orbitofrontal cortex; IFGtri, inferior frontal gyrus, triangular part; IFGop, inferior frontal gyrus, opercular part; tAV, anterior ventral thalamic nucleus.

The MCI cohort was therefore divided into two local structural subtypes: a thalamic-subcortical subtype (n = 190) and a frontal-paralimbic subtype (n = 161). After controlling for global neurodegenerative burden, the two subtypes showed distinct local anatomical deviation patterns (Fig. 1). The thalamic-subcortical subtype was characterized primarily by greater negative residual deviations in multiple thalamic nuclei and related subcortical regions, whereas the frontal-paralimbic subtype showed relatively greater involvement in cortical regions including the inferior frontal gyrus, anterior cingulate, orbitofrontal cortex, and parahippocampal areas. Notably, although the hippocampus showed strong discriminative value for disease staging in the full sample, it contributed little to subtype differentiation within MCI after accounting for global atrophy. Bootstrap resampling was performed to evaluate the robustness of the two-cluster solution. The adjusted Rand index (ARI) had a mean of 0.680 (SD = 0.225; median = 0.736), and the mean Jaccard index was 0.824 (SD = 0.160; median = 0.867), supporting the high reproducibility of the identified subtypes. This finding suggests a partial anatomical separation between regions indexing disease severity and regions capturing interindividual spatial variation.

### 3.4 Quantitative comparison of brain region features between subtypes

**Figure 2:**
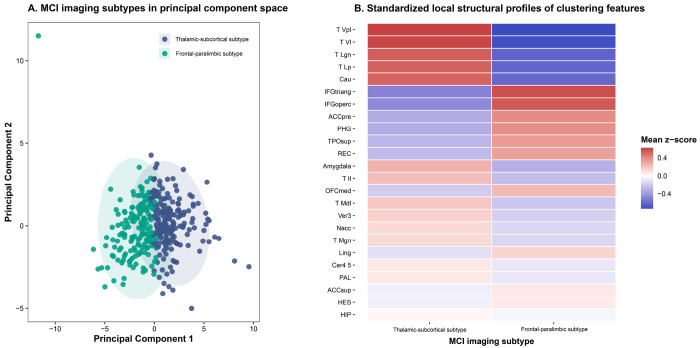
Structural characterization of MCI imaging subtypes in principal component space and across clustering features. (A) Distribution of participants with mild cognitive impairment (MCI) in the space defined by the first two principal components derived from local residual structural features. Each point represents one participant. Shaded ellipses indicate the dispersion of the two imaging subtypes identified by unsupervised clustering: the thalamic-subcortical subtype and the frontal-paralimbic subtype. (B) Heatmap showing the mean standardized local structural profiles (z-scores) of the clustering features across the two MCI imaging subtypes. Warmer colors indicate relatively higher residualized regional values, whereas cooler colors indicate relatively lower residualized regional values within each subtype. Abbreviations: T Vpl, thalamic ventral posterolateral nucleus; T Vl, thalamic ventrolateral nucleus; T Lgn, thalamic lateral geniculate nucleus; T Lp, thalamic lateral posterior nucleus; Cau, caudate nucleus; IFGtriang, triangular part of the inferior frontal gyrus; IFGoperc, opercular part of the inferior frontal gyrus; ACCpre, pregenual anterior cingulate cortex; PHG, parahippocampal gyrus; TPOsup, superior temporal pole; REC, gyrus rectus; Amygdala, amygdala; T Il, thalamic intralaminar nuclei; OFCmed, medial orbitofrontal cortex; T Mdl, thalamic mediodorsal lateral nucleus; Ver3, vermis lobule III; Nacc, nucleus accumbens; T Mgn, thalamic medial geniculate nucleus; Ling, lingual gyrus; Cer4 5, cerebellar lobules IV–V; PAL, pallidum; Accsup, supracallosal anterior cingulate cortex; HES, Heschl’s gyrus; HIP, hippocampus.

The two subtypes showed distinct distributions of local structural deviations across brain regions (Figure 2). Using standardized mean differences, the largest negative effects were observed in the ventral posterolateral nucleus (tVPL, SMD = −1.86), ventrolateral nucleus (tVL, SMD = −1.74), anteroventral nucleus (tAV, SMD = −1.63), lateral posterior nucleus (tLP, SMD = −1.56), and lateral geniculate nucleus (tLGN, SMD = −1.52), indicating stronger relative involvement of the thalamic system in the thalamic-subcortical subtype. In contrast, the largest positive effects were observed in the triangular part of the inferior frontal gyrus (IFGtriang, SMD = 1.19), opercular part of the inferior frontal gyrus (IFGoperc, SMD = 1.10), parahippocampal gyrus (PHG, SMD = 0.80), superior temporal polar region (TPOsup, SMD = 0.76), and pregenual anterior cingulate cortex (ACCpre, SMD = 0.68), indicating relatively greater cortical involvement in the frontal-paralimbic subtype. By comparison, the between-subtype difference in hippocampal volume was minimal (HIP, SMD = −0.05), further supporting the view that subtype-defining local variation was driven mainly by non-hippocampal structures once global atrophy burden had been accounted for (Figure 2).

### 3.5 Clinical relevance and additional explanatory value of local structural subtypes in MCI

Comparison of clinical phenotypes showed that the thalamic–subcortical subtype exhibited significantly greater cognitive impairment and poorer daily functioning than the frontal–paralimbic subtype, whereas differences in the neuropsychiatric measure (NPI) were modest. In regression models including age, sex, and years of education, and additionally controlling for global atrophy index (GAI), subtype labels remained significantly associated with both ADAS-Cog13 and FAQ. This suggests that these clinical differences are not simply attributable to overall brain atrophy.

**Figure 3:**
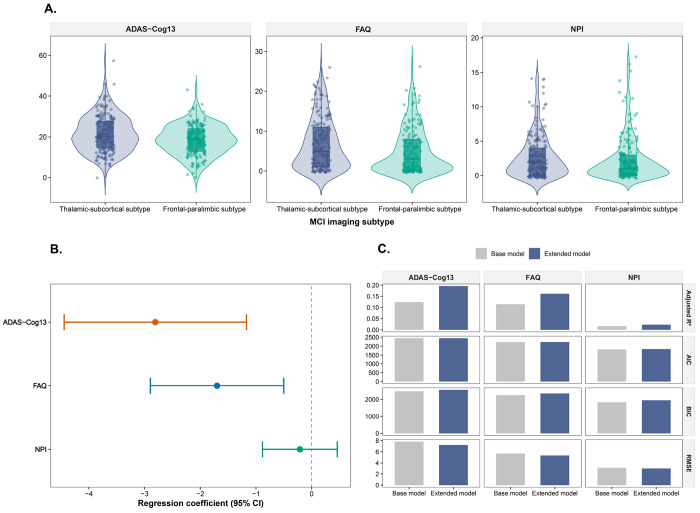
Clinical associations of MCI imaging subtypes and the added value of continuous local structural features. (A) Distributions of ADAS-Cog13, Functional Activities Questionnaire (FAQ), and Neuropsychiatric Inventory (NPI) scores across the two MCI imaging subtypes. Violin plots display the score distributions, with overlaid individual data points and embedded boxplots. (B) Adjusted associations between imaging subtype and clinical outcomes. Regression coefficients and 95% confidence intervals are shown for the comparison between the frontal-paralimbic subtype and the thalamic-subcortical subtype (reference), based on multivariable linear regression models adjusted for age, sex, years of education, and global atrophy index (GAI). Negative coefficients indicate lower clinical scores in the frontal-paralimbic subtype relative to the thalamic-subcortical subtype. (C) Comparison of base and extended models for each clinical outcome. The base model included imaging subtype together with age, sex, years of education, and GAI, whereas the extended model additionally included principal components derived from local residual structural features. Model performance is summarized using adjusted R^2^, Akaike information criterion (AIC), Bayesian information criterion (BIC), and root mean square error (RMSE).

Incremental model comparisons further indicated that continuous local structural variation provides additional explanatory value beyond discrete subtype labels. Specifically, adding principal components (PCs; k = 24, explaining ≥ 80% variance) of local residual structural features to the model containing subtype significantly improved model fit for ADAS-Cog13 (R^2^ = 0.162 to 0.217; ΔR^2^ = 0.055; FDR-adjusted p = 0.0446) and for FAQ (R^2^ = 0.144 to 0.197; ΔR^2^ = 0.052; FDR-adjusted p = 0.0446; Table 4). By contrast, the corresponding gain for NPI was small and non-significant (R^2^ = 0.029 to 0.056; ΔR^2^ = 0.027; FDR-adjusted p = 0.570). Together, these results indicate that, within MCI, local brain structure contains meaningful continuous inter-individual variability that is not fully captured by a discrete subtype framework. Integrating subtype labels with PC-based local structural features therefore improves characterization of cognitive and functional heterogeneity in MCI (Figure 3).

## Discussion

This study attempted to build a more rigorous hierarchical framework for the analysis of structural MRI data. The core aim was to clarify whether structural differences within the MCI population arise primarily from uneven disease severity or from true differences in local spatial patterns of brain involvement.(36, 37) The results showed that the GAI could effectively characterize overall neurodegenerative differences across the continuum from CN to MCI and AD. More importantly, even after strict statistical control for GAI, stable local brain structural subtypes could still be identified within the MCI group, most notably a thalamic-subcortical subtype and a frontal-paralimbic subtype. These subtypes not only showed specific spatial distribution patterns, but were also closely related to cognitive and functional performance. Taken together, the structural heterogeneity of MCI is reflected not only in differences in the overall degree of brain atrophy, but also in differences in the distribution of local brain involvement at similar levels of global burden.

When studying heterogeneity in neurodegenerative diseases, one of the most common methodological pitfalls is confounding by disease severity.(38, 39) In some previous imaging-based subtyping studies, high-dimensional anatomical features were directly entered into unsupervised algorithms.(23, 40, 41) Because most brain regions across the whole brain undergo some degree of atrophy as the disease progresses, this approach can lead clustering algorithms to capture mainly the overall burden of atrophy.(42) As a result, the identified subtypes may in fact become little more than labels for early, middle, and late disease stages. The residualization strategy used in this study offers one way to address this problem. By removing components that were highly collinear with global burden at an early stage, the resulting features more purely reflected the relative vulnerability or relative preservation of local brain regions. This approach not only improved the biological interpretability of the clustering results, but also confirmed that the clinical diversity of MCI is not determined only by the amount of brain atrophy.

Further analyses showed a relatively stable relationship between local structural subtypes and cognitive as well as daily functional outcomes, whereas the association with neuropsychiatric symptoms was weaker. This pattern is neurobiologically plausible. (43–45) In contrast, the mechanisms underlying neuropsychiatric symptoms are more complex. They often involve neurotransmitter imbalance at the functional network level, environmental stress, and the effects of physical comorbidities.(46, 47) Therefore, baseline structural imaging from a single time point has a natural upper limit in explaining behavioral and emotional abnormalities. It is worth noting that, in this study, the clear associations between subtype and ADAS-Cog13 and FAQ, together with the limited association with NPI, do not mean that local structural patterns are unrelated to neuropsychiatric symptoms. Rather, they more likely suggest that baseline structural MRI may have more limited explanatory power for such phenotypes than for cognition or daily functioning.

The additional explanatory value shown by continuous local structural information provides an important perspective for understanding the concept of “subtype.” As a dimensionality reduction product, subtype labels are useful because they provide a broad framework for population stratification and are convenient for clinical cohort selection and group comparison. However, converting information into discrete categories inevitably removes subtle differences among individuals within the same subtype.(48, 49) Compared with models that included subtype information alone, models that further included continuous local structural features showed better explanatory power for cognitive and functional outcomes.(50, 51) Therefore, in structural imaging subtyping research, subtype labels and continuous features should not replace each other. A more reasonable approach is to use them together. The former helps address stratification at the population level, while the latter helps explain the sources of individual-level differences.

In terms of specific local spatial patterns, after removing the effect of whole-brain atrophy, the two MCI subtypes were characterized by a thalamic-subcortical pattern and a frontal-paralimbic pattern rather than by differential involvement of the hippocampus alone. The thalamic-subcortical subtype showed greater residual reduction across multiple thalamic nuclei. These findings suggest that, once global atrophic burden is accounted for, heterogeneity within MCI is expressed more strongly in distributed cortico-subcortical systems than in the canonical medial temporal marker alone. The clinical differences between these two patterns are also informative. The thalamic-subcortical subtype showed worse cognitive performance and greater functional impairment, despite the fact that the subtype analysis had already adjusted for global atrophy burden. One possible interpretation is that additional vulnerability within thalamic-subcortical circuits may have disproportionate clinical consequences, because these regions are centrally involved in large-scale information relay, executive-attentional regulation, and cortico-subcortical integration. In contrast, the frontal-paralimbic subtype may represent a different axis of anatomical vulnerability that is not reducible to overall disease severity and may be associated with a different clinical expression rather than simply a more advanced stage. Importantly, the between-subtype difference in hippocampal structure was minimal, suggesting that hippocampal atrophy may function more as a shared marker of disease-stage burden across the AD continuum, whereas subtype-related heterogeneity within MCI emerges more clearly in non-hippocampal network components. This interpretation is consistent with the concept of selective vulnerability in neurodegeneration.(52–54) Even among individuals with a comparable level of whole-brain neurodegeneration, relatively greater involvement of distinct network nodes may shape different clinical profiles. These noncanonical patterns deserve further investigation in longitudinal and multimodal studies, particularly in relation to amyloid, tau, vascular burden, and network-level dysfunction.

This study also has several limitations. First, the sample was drawn from baseline data of ADNI-1. The cohort itself is somewhat selective, and its education level, health awareness, control of comorbidities, and imaging acquisition conditions still differ from those of real-world populations.(55) Therefore, the generalizability of the findings needs further validation in external samples. Second, the residualization framework used in this study was based on linear models. Although easy to interpret, linear residuals may not fully remove the global effect if nonlinear relationships or important interactions exist between local structure and global burden.(56) Third, to ensure data quality and clustering reliability, we excluded brain regions with high missing rates and combined bilateral homologous structures. While this stringent feature selection minimized noise and avoided imputation bias, it limited our ability to explore hemispheric asymmetry and may have omitted subtle pathological signals from smaller, excluded nuclei. Fourth, unsupervised clustering is still influenced by dimensionality reduction methods and distance measures. Although the present study showed good reproducibility of the subtypes through stability analyses, their boundaries are better understood as empirical stratification along a continuous disease spectrum rather than as fully discrete natural categories. Fifth, candidate ROI selection was first performed at the full-sample level based on disease-stage effects. This strategy reduced dimensional noise and increased pathological relevance, but it may also have excluded regions that are less strongly associated with the CN-to-AD gradient yet still informative for heterogeneity within MCI. Future studies could compare this approach with less constrained feature-selection strategies. Finally, this study was based on cross-sectional baseline data and therefore cannot directly assess the predictive value of these subtypes for the rate of cognitive decline, the trajectory of functional worsening, or the risk of progression to AD dementia. Future validation will require longitudinal follow-up data.

## Conclusion

In conclusion, heterogeneity in MCI cannot be understood solely in terms of the overall degree of brain atrophy. Using a hierarchical structural MRI framework, this study showed that local anatomical patterns remain detectable after accounting for global neurodegenerative burden and are meaningfully associated with cognition and daily functioning. Importantly, these local patterns are represented not only by discrete subtype assignments, which support population-level stratification, but also by continuous structural features that capture additional individual-level clinical variation. Together, these findings suggest that separating global burden from local structural organization may improve the biological interpretability of imaging heterogeneity in prodromal AD and may provide a useful framework for future precision stratification efforts.

## Supplementary Material

This is a list of supplementary files associated with this preprint. Click to download.

- supplement.docx

## Figures and Tables

**Table 1:** Baseline demographic, clinical, genetic, and global imaging characteristics across diagnostic groups

Characteristic	CN N = 205	MCI N = 351	AD N = 175	*p*-value
**Age, years, Median (Q1, Q3)**	77 (74, 80)	76 (71, 82)	78 (72, 83)	0.282
**Sex, n (%)**				**0.018**
Man	105 (51.2%)	219 (62.4%)	93 (53.1%)	
Female	100 (48.8%)	132 (37.6%)	82 (46.9%)	
**Handedness, n (%)**				0.372
Left	189 (92.2%)	321 (91.5%)	166 (94.9%)	
Right	16 (7.8%)	30 (8.5%)	9 (5.1%)	
**Education, years, Median (Q1, Q3)**	16.0 (14.0, 18.0)	16.0 (13.0, 18.0)	15.0 (12.0, 16.0)	**< 0.001**
**APOE ε4 allele, n (%)**				**< 0.001**
0	150 (73.2%)	161 (45.9%)	53 (30.3%)	
1	51 (24.9%)	147 (41.9%)	89 (50.9%)	
2	4 (2.0%)	43 (12.3%)	33 (18.9%)	
**ADAS-Cog13, Median (Q1, Q3)**	8 (5, 12)	20 (14, 26)	32 (26, 40)	**< 0.001**
**FAQ, Median (Q1, Q3)**	0 (0, 0)	4 (1, 10)	19 (12, 23)	**< 0.001**
**GAI, Median (Q1, Q3)**	−1.74 (−3.23, 0.10)	0.07 (−2.19, 2.09)	1.74 (0.01, 3.55)	**< 0.001**
**CSF volume, Median (Q1, Q3)**	381 (328, 435)	410 (361, 484)	438 (367, 510)	**< 0.001**
**Gray matter volume, Mean ± SD**	586 ± 52	575 ± 59	548 ± 57	**< 0.001**
**White matter volume, Median (Q1, Q3)**	480 (441, 524)	480 (436, 527)	463 (419, 504)	**0.008**
**TIV, Median (Q1, Q3)**	1,441 (1,355, 1,561)	1,475 (1,368, 1,573)	1,451 (1,320, 1,555)	0.117
**Mean cortical thickness, Median (Q1, Q3)**	2.29 (2.21, 2.37)	2.23 (2.13, 2.31)	2.17 (2.08, 2.25)	**< 0.001**

1. Data are presented as median (Q1, Q3), mean ± SD, or n (%), as appropriate.

2. Continuous variables were compared using the Kruskal–Walli’s test or one-way ANOVA, and categorical variables were 3.compared using Pearson’s chi- squared test.

4. GAI = global atrophy index; ADAS-Cog 13 = Alzheimer’s Disease Assessment Scale–Cognitive Subscale 13-item version; FAQ = Functional Activities Questionnaire; MMSE = Mini-Mental State Examination; TIV = total intracranial volume.

**Table 2:** Representative brain regions showing significant differences across cognitive status groups

Brain Region	CN, Mean ± SD	MCI, Mean ± SD	AD, Mean ± SD	Effect size (ε^2^)	FDR-corrected P	Direction
Hippocampus	35.216 ± 1.951	34.122 ± 2.073	32.757 ± 2.212	0.152	1.97×10^−23^	CN > MCI > AD
Medial geniculate nucleus (tMGN)	0.431 ± 0.084	0.400 ± 0.041	0.381 ± 0.042	0.150	2.53×10^−23^	CN > MCI > AD
Global atrophy index (GAI)	−1.489 ± 2.490	−0.031 ± 3.037	1.807 ± 3.030	0.147	4.60×10_−23_	CN < MCI < AD
Parahippocampal gyrus	32.503 ± 2.372	31.632 ± 2.022	30.716 ± 1.749	0.104	2.28×10^−23^	CN > MCI > AD
Anterior pulvinar nucleus (tPuA)	1.613 ± 0.815	1.730 ± 0.497	1.955 ± 0.565	0.075	4.61×10^−12^	CN < MCI < AD
Amygdala	9.195 ± 0.792	8.914 ± 0.838	8.718 ± 1.046	0.053	8.29×10^−9^	CN > MCI > AD
Medial orbitofrontal cortex	1.623 ± 0.350	1.729 ± 0.350	1.858 ± 0.428	0.049	3.67×10^−8^	CN < MCI < AD
Inferior frontal gyrus, triangular part	5.841 ± 1.290	5.867 ± 1.366	6.230 ± 1.331	0.016	2.69×10^−3^	CN < MCI < AD
Anterior ventral thalamic nucleus (tAV)	0.836 ± 0.188	0.838 ± 0.108	0.863 ± 0.108	0.016	2.94×10^−3^	CN < MCI < AD
Inferior frontal gyrus, opercular part	8.627 ± 1.187	8.693 ± 1.216	8.989 ± 1.199	0.016	2.94×10^−3^	CN < MCI < AD

1. Group differences were tested using the Kruskal–Wallis test. Effect size is reported as epsilon squared (ε^2^). Full results for all significant regions are shown in Supplementary Table S1.

2. Abbreviations: CN, cognitively normal; MCI, mild cognitive impairment; AD, Alzheimer’s disease; FDR, false discovery rate; ε^2^ = epsilon squared (effect size).

**Table 3:** Key neuroanatomical and clinical differences between MCI subtypes

Measure	S1 (n = 190), adjusted mean ± SE	S2 (n = 161), adjusted mean ± SE	Adjusted mean difference (S1 - S2)	Partial η^2^	FDR-adjusted *p* value
Ventral posterolateral thalamic nucleus	7.919 ± 0.042	6.872 ± 0.046	+1.047	0.461	1.02 × 10^−45^
Ventral lateral thalamic nucleus	12.634 ± 0.053	11.370 ± 0.058	+1.264	0.434	2.89 × 10^−43^
Pulvinar lateral nucleus	2.514 ± 0.033	1.837 ± 0.035	+0.677	0.389	3.67 × 10^−35^
Pulvinar anterior nucleus	1.970 ± 0.026	1.458 ± 0.028	+0.512	0.367	9.01 × 10^−33^
Pulvinar medial nucleus	16.166 ± 0.255	11.153 ± 0.276	+5.013	0.366	9.80 × 10^−33^
Anteroventral thalamic nucleus	0.893 ± 0.006	0.775 ± 0.007	+0.118	0.352	4.03 × 10^−32^
Lateral geniculate nucleus	1.874 ± 0.017	1.540 ± 0.019	+0.334	0.351	1.25 × 10^−31^
Lateral posterior thalamic nucleus	3.318 ± 0.062	2.155 ± 0.067	+1.163	0.346	1.95 × 10^−30^
Caudate	34.647 ± 0.180	31.358 ± 0.195	+3.289	0.309	2.57 × 10^−29^
Inferior frontal gyrus, triangular part	5.310 ± 0.069	6.493 ± 0.075	−1.183	0.257	2.49 × 10^−26^
Medial superior frontal gyrus	4.785 ± 0.108	6.549 ± 0.117	−1.764	0.244	1.61 × 10^−24^
Inferior frontal gyrus, opercular part	8.200 ± 0.067	9.255 ± 0.072	−1.055	0.231	3.13 × 10^−23^
ADAS-Cog 13	21.410 ± 0.573	18.604 ± 0.620	+2.806	0.040	0.002
FAQ	6.695 ± 0.420	4.997 ± 0.454	+1.698	0.028	0.008

Adjusted means were estimated from general linear models controlling for relevant covariates (e.g., age, sex, education, and intracranial volume/site, as applicable). Positive values of the adjusted mean difference indicate higher values in subtype 1 (S1), whereas negative values indicate higher values in subtype 2 (S2). P values were corrected for multiple comparisons using the false discovery rate (FDR) method. S1 = thalamic-subcortical subtype, S2 = frontal-paralimbic subtype.

**Table 4:** Nested model comparison within the MCI subgroup (Subtype and ROI-derived PCs)

Outcome	Model	n	R^2^	Adjusted R^2^	ΔR^2^ vs base	p (Subtype vs Base)	ΔR^2^ vs Base+Subtype	p (Extended vs Base+Subtype)	p_FDR (Extended vs Base+Subtype)	AIC	BIC
ADAS-Cog13	Base	351	0.134	0.124	–	–	–	–	–	2451.122	2474.2
	Base+Subtype	351	0.162	0.150	0.028	0.0008	–	–	–	2441.724	2468.7
	Extended	351	0.217	0.180	–	–	0.055	0.0167	0.0446	2439.700	2509.1
FAQ	Base	351	0.125	0.115	–	–	–	–	–	2228.470	2251.6
	Base+Subtype	351	0.144	0.132	0.019	0.0056	–	–	–	2222.643	2249.6
	Extended	351	0.197	0.158	–	–	0.052	0.0297	0.0446	2222.480	2291.9
NPI	Base	351	0.028	0.017	–	–	–	–	–	1814.987	1838.1
	Base+Subtype	351	0.029	0.015	0.001	0.5369	–	–	–	1816.598	1843.6
	Extended	351	0.056	0.011	–	–	0.027	0.5704	0.5704	1828.685	1898.1

Analyses were restricted to the MCI subgroup (n = 351). ΔR^2^ values indicate incremental variance explained in nested comparisons. p-values correspond to incremental (nested) model tests. FDR-adjusted p-values (BH) are reported for the Extended vs Base+Subtype step across outcomes. PCs: first 24 principal components explaining ≥ 80% cumulative variance.

## Data Availability

The datasets generated and/or analyzed in the current study are available in the figshare repository at: https://doi.org/10.6084/m9.figshare.32038887.

## Abbreviations

CN: cognitively normal
MCI: mild cognitive impairment
AD: Alzheimer’s disease
FDR: false discovery rate
ε^2^: epsilon squared (effect size)
